# The role of automated evaluation techniques in online professional translator training

**DOI:** 10.7717/peerj-cs.706

**Published:** 2021-10-04

**Authors:** Dasa Munkova, Michal Munk, Ľubomír Benko, Petr Hajek

**Affiliations:** 1 Department of Translation Studies, Constantine the Philosopher University in Nitra, Nitra, Slovakia; 2 Department of Computer Science, Constantine the Philosopher University in Nitra, Nitra, Slovakia; 3 Science and Research Centre, University of Pardubice, Pardubice, Czech Republic

**Keywords:** Online education, Automatic MT metrics, Residuals, Translator training, Post-editing, Formative assessment

## Abstract

The rapid technologisation of translation has influenced the translation industry’s direction towards machine translation, post-editing, subtitling services and video content translation. Besides, the pandemic situation associated with COVID-19 has rapidly increased the transfer of business and education to the virtual world. This situation has motivated us not only to look for new approaches to online translator training, which requires a different method than learning foreign languages but in particular to look for new approaches to assess translator performance within online educational environments. Translation quality assessment is a key task, as the concept of quality is closely linked to the concept of optimization. Automatic metrics are very good indicators of quality, but they do not provide sufficient and detailed linguistic information about translations or post-edited machine translations. However, using their residuals, we can identify the segments with the largest distances between the post-edited machine translations and machine translations, which allow us to focus on a more detailed textual analysis of suspicious segments. We introduce a unique online teaching and learning system, which is specifically “tailored” for online translators’ training and subsequently we focus on a new approach to assess translators’ competences using evaluation techniques—the metrics of automatic evaluation and their residuals. We show that the residuals of the metrics of accuracy (BLEU_n) and error rate (PER, WER, TER, CDER, and HTER) for machine translation post-editing are valid for translator assessment. Using the residuals of the metrics of accuracy and error rate, we can identify errors in post-editing (critical, major, and minor) and subsequently utilize them in more detailed linguistic analysis.

## Introduction

Translation is a specific skill, which has a specific place in the system of foreign language acquisition and is considered to be an additional value in foreign language mastery (Wrede, 2012a, 2012b).

Conventional education for translators mostly provides opportunities for the development of linguistic and translation competences or criteria for translation quality but, influenced by technological developments, language service providers have become more interested in those translators who are capable of utilizing their technological competencies together with the above-mentioned competences in the process of translation services (Ismail, Nasser Alsager & Omar, 2019). Doherty (2016) is convinced the aim of translator training is not only to achieve the translator competence necessary for the translation process but also to acquire the abilities to manage technological developments integrated into the process of translation.

Today’s digital and economic world calls for changes in the approaches necessary for translation activities as part of training or preparation itself (Gavrilenko, 2018). Human translators no longer assume the ‘leading role’ in translation settings increasingly shaped by technology (Öner, 2019). The work of translators has shifted to the virtual environment (Robinson, Lobo & Gutiérrez-Artacho, 2017). Therefore, many educational institutions are challenged and face the same dilemma as to whether they are sufficiently prepared to satisfy the needs and the platforms when providing the training necessary for the current translation market (Esfandiari, Shokrpour & Rahimi, 2019). The solution tends to intensify the implementation of linguistic and translation technologies together with well-prepared learning programs into translator training, to achieve highly professional translation services, including technological literacy. Marczak (2018) claims that translator education seems to require the revision of the long-standing theories of translation which are taught, *i.e.*, to be updated with elements pertaining to computerized translation.

## Research objectives

The study comprises two partially interrelated objectives. The first objective focuses on a proposed online system, specifically designed and created for effective online learning and training by taking into account the technologisation of the translation process, as required by the language and translation industry. This will show that even such specific teaching and learning as translator training can be done online, through a specially designed and developed virtual system. In the proposed online system OSTPERE, students (future translators) learn not only to translate a text into a mother tongue or foreign language, but also to assess the translation quality of classmates.

The second, main and subsequent objective introduces a new approach to assess translators’ competences through their post-edited machine translations (PEMTs) using automatic evaluation measures and residuals—by relying on computational analysis.

We were inspired by the automatic metrics of machine translation (MT) evaluation and residual analysis in our search for new approaches to the most objective evaluation of translation quality. In the same way as the performance of an MT system is evaluated to increase efficiency, we can assess translators’ performance during the processes of translation or post-editing (PE) in order to increase their work efficiency (translation quality).

The structure of the study is as follows: we provide a brief review of the related work focusing on teaching translation online. Then, we describe our proposed online system for translator training and an experiment with an innovative translator training model. The fifth section offers the results of translator training in terms of a formative assessment of post-editing as a process of translation. The next section provides a discussion of the results with a focus on the errors made by students during post-editing. The last section summarizes the contributions and limitations of the presented research.

## Literature review

The needs of the market focus attention on the following four fields: (1) Machine translation and post-editing. MT is not only a current instant fascination, but it is a homogenous component of the translation process and shapes the future of the translation market. Since MT itself without human intervention makes no sense, we already register the demand for PE services. (2) Video translation and/or video content translation (marketing and other) is growing considerably. It is estimated that 82% of internet transmission will be provided *via* video in the year 2021 (Drummond-Butt, 2019). As Drummond-Butt (2019) state 8.72% of clients prefer video guidance rather than written manuals. This again increases the demand not only for the translation service but also for subtitling. (3) Education and e-learning is also part of the demand. The Forbes journal predicts the e-learning market will reach 325 billion US dollars by the year 2025 (McCue, 2018). Nowadays, Massive Open Online Courses (MOOCs) cover the great need for educational training. Shah (2018) claims that more than 900 universities offered 11,400 MOOCs in 2018 and the number of people applying was 101 million. Gradually, they have become part of face-to-face teaching at universities, which were forced to transfer to virtual education in 2020 due to the pandemic. The situation with COVID-19 showed two main limitations of online educational content. The first was the high number of successful achievers of university education. The second problem was language barriers (Kordoni et al., 2016). There are a great number of educational online materials, but most of them are written in English which is why the demand for services of translation to other languages and the ability to use various translation technologies, such as MT, is increasing greatly. In 2015 the project for translation named Translation for MOOCs (TraMOOC) was designed. It eliminated the language barrier in online educational contexts and at the same time created a very specific environment for research in online educational content. Based on the results of manual and automatic evaluations of the machine translation quality of MOOC contents, the used MT systems were optimized (Sennrich et al., 2017). Moreover, PE appeared to be a very successful solution for quality achievements including trust among the final users (Hu, O’Brien & Kenny, 2020). Apparently, MT and PE introduce successful solutions for effective online educational content. (4) Business globalization and the virtual market (e-commerce and online content) is the last of the fields where the above-mentioned areas meet or intersect. Although this direction does not introduce worries or transform all translators into post-editors, it is important to focus on the preparation for such a situation and to prepare the innovation of curricula for students of translation studies in order to provide those competences and abilities, which will lead them to professional positions in the globalized world.

Although module technologies have become widespread in the environment of educational institutions, they are usually offered in the form of module and/or learning packages and learning exercises (Zaidan & Callison-Burch, 2011). However, translation studies education also requires special training in translation. As Gorozhanov, Kosichenko & Guseynova (2018) set out, there are three basic principles of online translator training, firstly, accepting the teacher as an expert in the field, secondly, accepting the teacher as a mediator between the students and the online environment, and thirdly, the accessibility of communication channels for regular consultations between the students and the teacher-expert.

Xu (2021) suggested an assistant platform for teaching Japanese translation *via* the virtual platform Virtualenv and the programming language Python. The platform was tested on a pharmacological text of 15,000 words and compared with the auxiliary translation effect. Da’u & Salim (2019) have implemented a part-of-speech tag embedding channel into a deep convolutional neural network to improve the sentiment analysis. The results of using the tags indicated better performance when compared to the baseline models. Wei (2020) applied artificial intelligence to problem-solving situations in translation. He examined a simple English-Chinese MT system to evaluate the level of computer-aided translation when teaching translation. He tried to find possible translations (sentences) using selected key words from the English sentences. He concluded that it is possible to decrease the study time for learning translation and increase teaching efficiency. It corresponds to his previous research (Wei, 2021), where he used an educational big data platform (cloud) and the WeChat application for web-assisted translation teaching and blended learning to teach English translation at Chinese universities (Wei, 2021). Wei (2021) showed a significant difference in increasing the translation level of students being trained using such a blended learning method.

The above-mentioned studies mainly deal with the translation itself and do not offer the teacher better control over students’ performance or students’ progress in translation.

## Ostpere as a translation system for teaching and training translation

We have designed and created a unique interactive online system—OSTPERE—for teaching translation (OSTPERE—Online System for Translation, Post-editing, Revision and Evaluation). The system was originally designed to teach MT and PE (Munková, Kapusta & Drlík, 2016; Munková et al., 2020), but due to the situation related to COVID-19 (face-to-face teaching also had to be moved to the online environment) the system was modified for the needs of teaching human translation and revision. The system was created using CodeIgniter, the PHP framework, and a MySQL database was used for data storage. For better orientation in the system-user relationship or human-machine interaction, functional requirements have been defined for the designed system to provide support for the teacher of the given subject:

- Uploading and processing files with text aligned in xls format;
- Documents selection for PE;
- Documents selection for translation (human translation, HT);
- Revision of PEMTs and human-translated documents.

**Figure 1:**
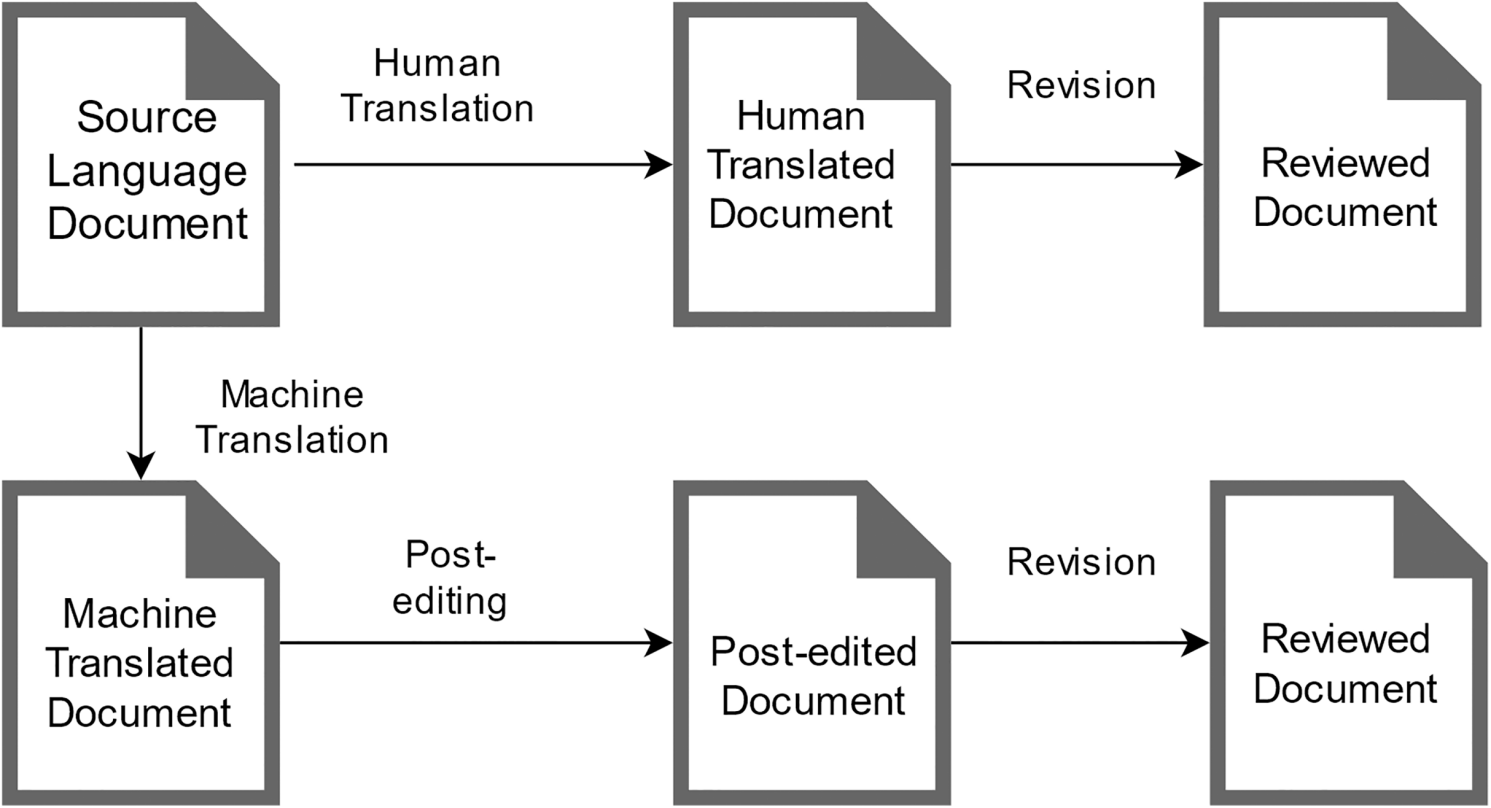
Workflow of teaching and learning translation/post-editing with the OSTPERE system during the lesson.

**Figure 2:**
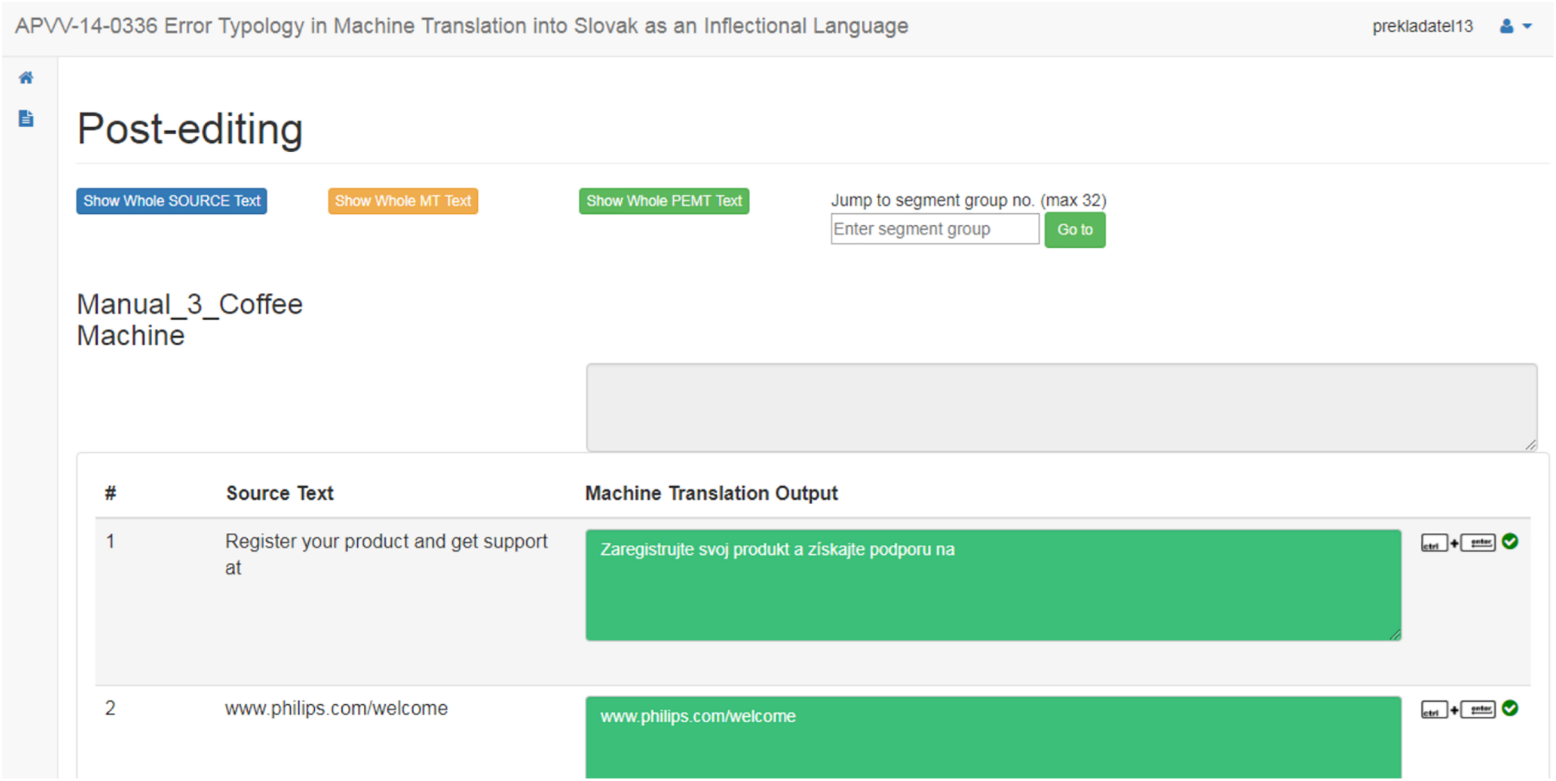
Interface for learning/teaching translation or post-editing.

The system offers an online interface available 24/7 using a web browser. This ensures flexibility in access to assignments for students as well as for teachers to review and assess students’ assignments (Fig. 1). In the OSTPERE system, human translation and PE are conducted by students (Fig. 2) and later mutually proofread and revised by students (classmates), and finally by the teacher. The data from the website is saved into the MySQL database and linked to each other. The source text is connected to the machine translation through a foreign key. Based on this connection the student during post-editing sees both source and machine translated segment. The post-edited segment is then saved to a new table that contains the information about the machine translated segment again through a foreign key. The data is then exported using a SQL query based on the requirements of the analysis.

Unlike existing systems and platforms, the OSTPERE system links the principles of professional systems and tools used in the translation industry (*e.g.*, Trados) with the needs of teaching translation. It simulates the work within the professional systems but also has implemented elements for the formative assessment of students, which allows the teachers to better focus on more complex sentences. The procedure of text processing within the OSTPERE system is as follows (Fig. 1):

1. Any formatting is deleted from the obtained textual document in the source language (in a foreign language);
2. Textual document is translated into the target language (Slovak) using an MT engine (e-translation and Google Translate API) (Google, 2016);
3. The source text is aligned with the MT output using the HunAlign tool (Varga et al., 2005), resulting in a data matrix containing the text segments;
4. The data matrix is uploaded to the OSTPERE system;
5. In the OSTPERE system, human translation and PEMT are conducted by students (Fig. 2);
6. The human translation and the PEMT are mutually proofread and revised by students and finally by the teacher.

## Materials & methods

Our purpose is to show that even such specific teaching and learning as translator training can be conducted interactively and online, through a specially designed and developed virtual system. In addition, the assessment of student translation competence can be more objective using automatic evaluation metrics, and the results of students’ assessments can provide us with a better understanding of what students know and what is difficult for them when training on translation or PE (using automatic metrics and residuals). For this purpose, an experiment consisting of three subtasks was conducted. The first was to verify the validity of the residuals of automatic evaluation metrics for student assessment. The second determined the boundary of translation errors and identified the redundant residuals of automatic evaluation metrics, and the last identified the segments with minor, major, and critical errors.

### Participants

The experiment was attended by 20 students (3 men and 17 women, with an average age of 23 years) of master’s degrees in translation studies. In the academic year 2019/2020, they enrolled in an MT and PE course (13 weeks). After completing the introductory information about MT and the basic PE rules or guidance, the students had the task of translating one selected journalistic text in the online system, to post-edit one MT text and review two texts (HT and PEMT) produced by their classmates. Students translated from the foreign language into their mother tongue—Slovak. The errors found were analysed during the next lesson.

### Dataset

After completing the course, we obtained a data matrix consisting of 3,192 segments from 156 documents. The average length of a source segment is 17 words and in the case of translated and PEMT texts, 15 words (see Data Matrix).

### Automatic evaluation metrics

In general, there are two approaches to evaluating MT quality. The first is based on human evaluation and the second relies on methods and techniques that automate this evaluation. We focused on automatic metrics, but we also used the post-editing method, which is a manual evaluation of MT quality. According to the criterion of concordance, we selected automatic metrics based on lexical concordance and divided them into metrics of accuracy and metrics of error rate (Munk, Munkova & Benko, 2018).

Metrics of accuracy are based on the closeness of the MT output/hypothesis (h) with the reference (r) in terms of n-grams; they calculate their lexical overlap in (A) the number of common words ( $h \cap r$); (B) the length (number of words) of MT output and (C) the length (number of words) of the reference (Benkova et al., 2021). The higher the values of these metrics, the higher the translation quality (Benkova et al., 2021).

BLEU is a geometric mean of n-gram precisions ( ${\rm p = }{{\rm A}/{\rm B}}$) and a brevity penalty (BP), *i.e.*, length-based penalty to prevent very short sentences as compensation for inappropriate translation.

$$BLEU{\rm \; }\left( n \right) = exp\mathop \sum \limits_{n = 1}^N {w_n}\log {p_n} \times BP,$$

where ${W}_{{n}}$ are weights for different.

$$BP = \left\{ {\matrix{1,  {if}  r > r \cr {e^{1 - {r \over h}}}, if & h \le r}} \right.,$$

where *r* is reference translation of hypothesis *h*.

*BLEU* represents two features of translation quality—*adequacy* and *fluency* by calculating words or lexical *precision* (Munk, Munkova & Benko, 2018).

**Metrics of error rate** are based on edit distance; they calculate the Levenshtein distance between an MT output/hypothesis (*h*) and a reference/human translation (*r*). The higher the values of these metrics, the lower the translation quality.

**WER** is based on the edit distance (edit operations) and does not permit reordering of words.

$WER\left( {h,r} \right) = {{mi{n_{e \in E\left( {h,r} \right)}}\left( {I + D + S} \right)} \over {\left| r \right|}}$, where *I* is the number of adding words, *D* is the number of dropping words, *S* is the number of replacements (in sequence or path *e*), and $mi{n_{e \in E\left( {h,r} \right)}}$ is a minimal sequence of adding, dropping and replaced words necessary to transform the MT output (*h*) into the reference (*r)* (Munk & Munkova, 2018).

**PER** is based on *WER* but ignores the ordering of the words in a sentence (Munk, Munkova & Benko, 2018).

**CDER** (Cover Disjoint Error Rate) (Leusch, Ueffing & Ney, 2006; Munk, Munková & Benko, 2016) is a measure oriented towards *recall* but based on the Levenshtein distance.

$CDER\left( {h,r} \right) = {{mi{n_{e \in E\left( {h,r} \right)}}\left( {I + D + S + long\; jump\left( e \right)} \right)} \over {\left| r \right|}},$ where *long jump* (*e*) is the number of long jumps.

**TER** (Translation Error Rate) (Snover et al., 2006) is based on the edit distance like the WER metric. The difference between them is that the TER allows block movement of words - shift.

$TER\left( {h,r} \right) = {{mi{n_{e \in E\left( {h,r} \right)}}\left( {I + D + S + shift\left( e \right)} \right)} \over {\left| r \right|}},$ where *shift* (*e*) is the number of shifts.

**HTER** (Human-mediated Translation Error Rate) (Snover et al., 2006) is built on TER and human judgments. Humans do not assess machine translation directly but they generate a new reference (PEMT) according to which the translation errors are calculated.

In our case, we have two references: (1) a revised PEMT (gold standard), which was used to calculate the HTER metric for PEMT and MT, and (2) a revised HT (gold standard), which was used to calculate the remaining metrics of error rate (PER, WER, TER, and CDER) and accuracy (BLEU_n) for PEMT and MT.

### Residuals

Errors and residuals are closely related measures of deviation (Munk & Munkova, 2018). The error is the deviation of the observed value (PEMT/reference) from the expected value (MT), while the residual of the observed value is the difference between the observed and predicted value of the quality (Munk & Munkova, 2018; Drlik & Munk, 2019)

$$Residual = Observed - Predicted.$$

Residuals can help us to assess the adequacy of the model, in our case the PE quality. For assessing if the data fit the model and whether the model is useful, a residual analysis is one of the effective methods. According to Bollen & Arminger (1991), the unstandardized residuals can be plotted to help identify unusual values of the residuals in comparison to others, while the calculated values of residuals depend on the metric used to measure the observed variables, in our case, metrics of automatic MT evaluation such as metrics of accuracy and error rate (Munk & Munkova, 2018).

Munk & Munkova (2018) claim that evaluation of MT output can be viewed as an evaluation of the validity of assumptions of a statistical model. Residuals allow us to identify patterns, better understand and interpret problems of the model and subsequently eliminate, correct, analyse them or analyse their influence on the quality of post-editing (*e.g.*, mistakes in post-editing).

We used residuals to compare the scores of automatic metrics of PEMT with MT at the sentence level based on (Munk & Munkova, 2018). In our case, the analysis composed of residual analysis is defined as follows

$${\left( {residual{\rm \; }value} \right)_i} = {\left( {score{\rm \; }of{\rm \; }PE{\rm \; }sentence} \right)_i} - {\left( {score{\rm \; }of{\rm \; }MT{\rm \; }sentence} \right)_i},\;\; i = 1,2, \ldots ,I,$$

while *I* is a number of examined sentences in the dataset and *PE* means post-edited (Munk & Munkova, 2018).

To identify redundant metrics, it was necessary to create a method to compare the individual metrics of error rate and accuracy for each error category. To calculate the degree of concordance in segments identification we proceeded as follows

$${R_{error}} = \displaystyle{{CoS\left( {metric{A_{error}}\; AND\; metric{B_{error}}} \right)} \over {CoS\left( {metric{A_{error}}\; OR\; metric{B_{error}}} \right)}},$$

where *R* is the degree of concordance in the identification of the segments based on the residuals of the metrics of error rate (or accuracy), the *error* corresponds to an individual type of identified error (minor, major or critical error), *CoS* is the Count of Segments, *metricA* and *metricB* are residuals of two different error rate (or accuracy) metrics for which we identify the degree of concordance. We determine the proportion of segments, in which the residuals of the two examined metrics of error rate (or accuracy) determine the same type of error against all segments of the given metrics with the identified type of error.

## Results

### The validity of the automatic evaluation metrics

The following assumptions were formulated about the validity of selected metrics:

– Residuals of metrics of error rates (PER, WER, HTER, TER, and CDER) of the PEMT and MT output are valid for measuring students’ translation competences.
– Residuals of metrics of accuracy (BLEU_1, BLEU_2, BLEU_3, and BLEU_4) of the PEMT and MT output are valid for measuring students’ translation competences.

The evaluation from the final state examination—Translation (PT)—was selected as a valid criterion.

Due to the violation of the sphericity assumption (an assumption about the structure of the covariance matrix in a repeated measures design), we will use adjusted tests for repeated measurements to test the null statistical hypotheses resulting from the defined assumptions.

H0: *Residuals of PEMT and MT output do not depend on the metric of error rate/accuracy nor on the combination of these metrics with PT assessment *i.e.*, the interaction within-group and between-groups factors*.

#### The validity of the error rate metrics

We used the Mauchly Sphericity test (*W* = 0.008; *Chi-Square* = 15,333.936; *df* = 9; *p* = 0.0000) to verify the assumption of using the analysis of variance for repeated measures with five levels (residuals of PER, WER, HTER, TER, and CDER metrics of the error rate of PEMT and MT output). In our case, the test is significant ( $p < 0.001$), *i.e.*, the assumption is violated. If the condition of sphericity of the covariance matrix is not met, error type I increases. Therefore, we adjust the degrees of freedom for the used *F*-test using Greenhouse-Geisser and Huynh-Feldt adjustments (Table 1) to achieve the declared level of significance.

**Table 1:** Adjusted tests for residuals of error rate metrics of PEMT and MT output.

	G-G	G-G	G-G	G-G	H-F	H-F	H-F	H-F
	Epsilon	Adj.df1	Adj.df2	Adj. *p*	Epsilon	Adj.df1	Adj.df2	Adj. *p*
Err_PE-MT	0.522	2.086	6,650.817	0.0000	0.522	2.090	6,661.521	0.0000
Err_PE-MT *Ass_PT	0.522	6.259	6,650.817	0.0000	0.522	6.269	6,661.521	0.0000

**Note:**

Err_PE-MT, residuals of error rate metrics for PEMT and MT output; Ass_PT, PT assessment; G-G, Greenhouse-Geisser adjustment; H-F, Huynh-Feldt adjustment; Epsilon, test statistic; df, degrees of freedom; *p*, probability value.

The results are identical (Table 1). We reject the null hypothesis at the 0.1% significance level, *i.e.*, the error rate metrics and the combination of factors (metric and PT) do not affect the residual values of the PEMT and MT output.

Multiple comparisons for between-groups factor (Ass_PT) show that the residuals of the error rate of PEMT and MT output distinguish well between students with grade E and better, as well as between students with grade A and C (Table 2). On the contrary, residuals cannot distinguish between students with grade A and B or between students with B and C.

**Table 2:** Multiple comparisons (Tukey HSD test for unequal N) for the PT assessment.

Ass_PT	PE-MT	1	2	3
A	−0.2358	****		
B	−0.1893	****	****	
C	−0.1801		****	
E	−0.1290			****

**Note:**

Ass_PT, PT assessment; PE-MT, mean of residuals of PEMT and MT output; 1/2/3, homogeneous groups (HG); **** HG component.

If we look at the results in terms of within-group factor, *i.e.*, individual error rate metrics, the residuals of the error rate metrics WER, TER, and HTER of the PEMT and MT output assess on average the PE quality at the same level (Table 3), likewise the metrics HTER and PER (Table 3).

**Table 3:** Multiple comparisons (Tukey HSD test) for the residuals of error rate metrics of PEMT and MT output.

Err_PE-MT	PE-MT	1	2	3
WER_PE-MT	−0.1824	****		
TER_PE-MT	−0.1824	****		
HTER_PE-MT	−0.1820	****	****	
PER_PE-MT	−0.1778		****	
CDER_PE-MT	−0.1644			****

**Note:**

Err_PE-MT, residuals of error rate metrics of PEMT and MT output; PE-MT, mean of residuals of PEMT and MT output; 1/2/3, homogeneous groups (HG); **** HG component.

On the contrary, the metric CDER (Table 3) seems to be the most stringent of all examined error rate metrics. In this case, the PE error rate was closest to the MT error rate, which is also confirmed by the univariate results (Tables 4–6).

**Table 4:** Multiple comparisons (Tukey HSD test for unequal *N*) for residuals of (a) PER and (b) WER of PEMT and MT output.

Ass_PT	PER_PE-MT	1	2	Ass_PT	WER_PE-MT	1	2
A	−0.2169	****		A	−0.2431	****	
B	−0.1871	****		B	−0.1954	****	
C	−0.1825	****		C	−0.1851	****	
E	−0.1366		****	E	−0.1289		****

**Note:**

Ass_PT, PT assessment; PER_PE-MT/WER_PE-MT, mean of PER/WER residuals of PEMT and MT output; 1/2, homogeneous groups (HG); **** HG component.

**Table 5:** Multiple comparisons (Tukey HSD test for unequal *N*) for residuals of (a) HTER and (b) TER of PEMT and MT output.

Ass_PT	HTER_PE-MT	1	2		Ass_PT	TER_PE-MT	1	2
A	−0.2431	****			A	−0.2431	****	
B	−0.1940	****			B	−0.1948	****	
C	−0.1858	****			C	−0.1858	****	
E	−0.1289		****		E	−0.1289		****

**Note:**

Ass_PT, PT assessment; HTER_PE-MT/TER_PE-MT, mean of HTER/TER residuals of PEMT and MT output; 1/2, homogeneous groups (HG); **** HG component.

**Table 6:** Multiple comparisons (Tukey HSD test for unequal *N*) for residuals of CDER of PEMT and MT output.

Ass_PT	CDER_PE-MT	1	2	3
A	−0.233		****	
B	−0.175	****		
C	−0.161	****		
E	−0.122			****

**Note:**

Ass_PT, PT assessment; CDER_PE-MT, mean of CDER residuals of PEMT and MT output; 1/2/3, homogeneous groups (HG); **** HG component.

The residuals of the metrics PER, WER, HTER and TER of the PEMT and MT output (Tables 4 and 5) have, on average, the same distinguishing ability, where all four distinguish well between students with grade E and better.

On the contrary, they do not distinguish between students with grades A and students with grade B or C. The residuals of the metric CDER of PEMT and MT output (Table 6) distinguish well not only students with grade E and better but also students with grade A and worse. On the contrary, they do not distinguish between students with grades B and C.

#### Validity of the accuracy metrics

We proceeded similarly with accuracy metrics: (a) we verified the assumption of using an analysis of variance for repeated measures with four levels (residuals of accuracy metrics BLEU_1, BLEU_2, BLEU_3, and BLEU_4 of PEMT and MT output), (b) due to violation of the assumption of sphericity of the covariance matrix (*W* = 0.295; *Chi-Square* = 3889.888; *df* = 5; *p* < 0.001) we used adjusted tests (Greenhouse-Geisser and Huynh-Feldt adjustments) to test null statistical hypotheses, (c) we rejected the null hypotheses at the 0.1 % significance level, claiming that accuracy metrics (*GG*: *Epsilon* = 0.588; *df1* = 1.765; *df*2 = 5626.588; *p* < 0.001) and a combination of factors (metric and PT assessment) do not affect the residual values of the PEMT and MT output (GG: *Epsilon* = 0.588; *df1* = 5.295; *df*2 = 5626.588; *p* < 0.001).

Multiple comparisons for the between-groups factor (Ass_PT) show (Table 7A) that the residuals of accuracy metrics of PEMT and MT output achieve on average the same distinguishability as the error rate metrics (Table 2), *i.e.*, they distinguish well students with grade E and better, as well as students with grades C and A. On the contrary, they cannot distinguish between students with grades C and B as well as between students with grades B and A. However, in terms of the within-group factor (Table 7B), *i.e.*, individual metrics of accuracy, the results are different (Table 3). Residuals of the accuracy metrics BLEU_1, BLEU_2, BLEU_3, and BLEU_4 of the PEMT and MT output (Table 7B) assess the PE quality at different levels (there are statistically significant differences in the values of the residuals of the metrics of accuracy). As expected, the metric BLEU_4 (Table 7B) is the most stringent of all examined metrics of accuracy and therefore it also has the best distinguishability, which is also confirmed by the univariate results (Tables 8 and 9).

**Table 7:** Multiple comparisons (a) Tukey HSD test for unequal N for the PT assessment (b) Tukey HSD test for unequal *N* for residuals of accuracy metrics of PEMT and MT output.

Ass_PT	PE-MT	1	2	3	Cor_PE-MT	PE-MT	1	2	3	4
E	0.0998			****	BLEU_4_PE-MT	0.1290	****			
C	0.1551	****			BLUE_3_PE-MT	0.1522		****		
B	0.1741	****	****		BLEU_1_PE-MT	0.1687			****	
A	0.2186		****		BLEU_2_PE-MT	0.1756				****

**Note:**

Ass_PT, assessment of subject Translation; Cor_PE-MT, residuals of accuracy metrics of PEMT and MT output; PE-MT, mean of residuals of PEMT and MT output; 1/2/3/4, homogeneous groups (HG); **** HG component.

**Table 8:** Multiple comparisons (Tukey HSD test for unequal *N*) for residuals of (a) BLEU_1 and (b) BLEU_2 metrics of the accuracy of PEMT and MT output.

Ass_PT	BLEU_1_PE-MT	1	2	Ass_PT	BLEU_2_PE-MT	1	2	3
E	0.1253		****	E	0.1179			****
C	0.1714	****		C	0.1781	****		
B	0.1792	****		B	0.1891	****	****	
A	0.2155	****		A	0.2462		****	

**Note:**

Ass_PT, assessment of subject Translation; BLEU_1_PE-MT/BLEU_2_PE-MT, mean of residuals of metrics BLEU_1/BLEU_2 of PEMT and MT output; 1/2/3, homogeneous groups (HG); **** HG component.

**Table 9:** Multiple comparisons (Tukey HSD test for unequal *N*) for residuals of (a) BLEU_3 and (b) BLEU_4 metrics of the accuracy of PEMT and MT output.

Ass_PT	BLEU_3_PE-MT	1	2	3	Ass_PT	BLEU_4_PE-MT	1	2	3
E	0.0898			****	E	0.0663		****	
C	0.1479	****			C	0.1230			****
B	0.1731	****	****		B	0.1548	****		
A	0.2258		****		A	0.1868	****		

**Note:**

Ass_PT, assessment of subject Translation; BLEU_3_PE-MT/BLEU_4_PE-MT, mean of residuals of metrics BLEU_3/BLEU_4 of PEMT and MT output; 1/2/3, homogeneous groups (HG); **** HG component.

Residuals of the metric BLEU_1 (Table 8A) have the worst distinguishability, they only distinguish well students with grade E and better. Residuals of the metrics BLEU_2 and BLEU_3 of the PEMT and MT output (Tables 8B and 9A) have, on average, the same distinguishability. Both metrics distinguish well between students with grade E and better assessments and between students with grades C and A. On the contrary, they do not distinguish between students with grades C and B as well as between students with grades B and A. Residuals of the metric BLEU_4 of the PEMT and MT output (Table 9B) distinguish well not only between students with grade E and better but also between students with grade C and better. On the contrary, they do not distinguish between students with grades B and A.

### Identification of PE errors based on residuals of error rate and accuracy metrics of PEMT and MT output

The results of descriptive statistics (Tables 10 and 11) show that the current quality of MT output is at a satisfying level (MT system works quite well). The median of the residuals of the error rate metrics as well as the accuracy metrics of the PEMT and MT output is close to 0. In the case of the residuals of the error rate metrics (Table 10), a small but negative value of the median was achieved, indicating the expected lower error rate of the PEMT against MT output. Similarly, for the residuals of the metrics BLEU_1 and BLEU_2 (Table 11), a small but positive value was achieved, indicating the expected higher accuracy of the PEMT. Unexpected results are the residual values of the metrics BLEU_3 and BLEU_4 (Table 11), where the median is equal to 0, which indicates approximately the same quality of the PEMT and MT output. Based on the calculated characteristics (Tables 10 and 11), we set the error boundary (limits). In the case of residuals of the error rate/accuracy metrics of the PEMT and MT output, a minor error is given by the value of the *upper/lower quartile*, which reaches the value 0. It means that if the PEMT achieves a higher error rate (Table 10) or less accuracy (Table 11) against the MT output, we consider this error to be minor. To identify the major error in case of error, we used the *Upper Quartile + Quartile Range* limit (Table 10) and in case of accuracy *Lower Quartile – Quartile Range* (Table 11). If the residuals value of the error rate/accuracy metrics is extreme, *i.e.*, higher than *Upper Quartile +* 1.5 *Quartile Range* (Table 10), or less than *Lower Quartile –* 1.5 *Quartile Range* (Table 11), we consider this error to be critical.

**Table 10:** Error limits of the error rate metrics of the PEMT and MT output.

	*N*	Median	Min	Max	Lower Quartile	Minor Error	Major Error	Critical Error	Range	Quartile Range
PER_PE-MT	3192	−0.111	−1.000	0.445	−0.272	**0.000**	**0.272**	**0.408**	1.445	0.272
WER_PE-MT	3192	−0.100	−1.000	0.667	−0.272	**0.000**	**0.272**	**0.408**	1.667	0.272
HTER_PE-MT	3192	−0.100	−1.000	0.667	−0.272	**0.000**	**0.272**	**0.408**	1.667	0.272
TER_PE-MT	3192	−0.100	−1.000	0.667	−0.272	**0.000**	**0.272**	**0.408**	1.667	0.272
CDER_PE-MT	3192	−0.100	−1.000	0.500	−0.250	**0.000**	**0.250**	**0.375**	1.500	0.250

**Note:**

Limit values are marked in bold, Minor Error > *Upper Quartile*, Major Error > *Upper Quartile + Quartile Range*, Critical Error > *Upper Quartile +* 1.5*Quartile Range*.

**Table 11:** Error limits of the accuracy metrics of the PEMT and MT output.

	*N*	Median	Min	Max	Minor Error	Upper Quartile	Major Error	Critical Error	Range	Quartile Range
BLEU_1_PE-MT	3,192	0.110	−0.340	1.000	**0.000**	0.250	**−0.250**	**−0.375**	1.340	0.250
BLEU_2_PE-MT	3,192	0.080	−0.500	1.000	**0.000**	0.250	**−0.250**	**−0.375**	1.500	0.250
BLUE_3_PE-MT	3,192	0.000	−0.550	1.000	**0.000**	0.190	**−0.190**	**−0.285**	1.550	0.190
BLEU_4_PE-MT	3,192	0.000	−0.550	1.000	**0.000**	0.110	**−0.110**	**−0.165**	1.550	0.110

**Note:**

Limit values are marked in bold, Minor Error < Lower Quartile, Major Error < Lower Quartile - Quartile Range, Critical Error < Lower Quartile - 1.5Quartile Range.

Descriptive characteristics of residuals indicate that some metrics may be redundant, especially in the case of residuals of error rate metrics.

Residuals of the metrics WER, HTER, and TER have a 99% match in identifying segments with minor errors (Table 12A) and a 100% match in identifying segments with major (Table 12B) and critical errors (Table 12C). Residuals of the metric PER achieve a 100% match with the above-mentioned metrics only in the identification of the segments of a critical error (Table 12C). Residuals of the metrics WER and TER are redundant in our case, *i.e.*, for post-editing errors identification, we will use not only residuals of the metrics PER and CDER, but also residuals of the metric HTER, which, in other cases, may have greater informative value, compared to residuals of the metrics WER and TER, for another reference. In the cases where no revised PEMT is available, it is possible to use the residuals of the metrics WER or TER, which have a very similar informative value.

**Table 12:** Matches in segment identification based on residuals of error rate metrics of PEMT and MT output with (a) minor, (b) major, and (c) critical error.

PE-MT	PER	WER	HTER	TER	CDER	PER	WER	HTER	TER	CDER	PER	WER	HTER	TER	CDER
PER		0.44	0.44	0.44	0.37		0.36	0.36	0.36	0.25		**1.00**	**1.00**	**1.00**	0.00
WER	0.44		**0.99**	**0.99**	0.45	0.36		**1.00**	**1.00**	0.27	**1.00**		**1.00**	**1.00**	0.00
HTER	0.44	**0.99**		**0.99**	0.45	0.36	**1.00**		**1.00**	0.27	**1.00**	**1.00**		**1.00**	0.00
TER	0.44	**0.99**	**0.99**		0.45	0.36	**1.00**	**1.00**		0.27	**1.00**	**1.00**	**1.00**		0.00
CDER	0.37	0.45	0.45	0.45		0.25	0.27	0.27	0.27		0.00	0.00	0.00	0.00	

**Note:**

Values higher than 0.9 are marked in bold, PE-MT PER/WER/HTER/TER/CDER, residuals of error rate metrics of PEMT and MT output.

Residuals of the metrics BLEU_1, BLEU_2, BLEU_3, and BLEU_4, in the identification of segments with minor errors (Table 13A), achieve a match less than 50%, in the case of a major and critical error (Tables 13B and 13C) less than 30%. Residuals of accuracy metrics are not redundant.

**Table 13:** Matches in segment identification based on residuals of accuracy metrics of PEMT and MT output with (a) minor, (b) major, and (c) critical error.

PE-MT	BLEU1	BLEU2	BLEU3	BLEU4	BLEU1	BLEU2	BLEU3	BLEU4	BLEU1	BLEU2	BLEU3	BLEU4
BLEU1		0.32	0.17	0.10		0.14	0.00	0.00		0.00	0.00	0.00
BLEU2	0.32		0.39	0.20	0.14		0.00	0.00	0.00		0.00	0.00
BLEU3	0.17	0.39		0.43	0.00	0.00		0.27	0.00	0.00		0.25
BLEU4	0.10	0.20	0.43		0.00	0.00	0.27		0.00	0.00	0.25	

**Note:**

PE-MT BLEU1/BLEU2/BLEU3/BLEU4, residuals of accuracy metrics of PEMT and MT output.

**Figure 3:**
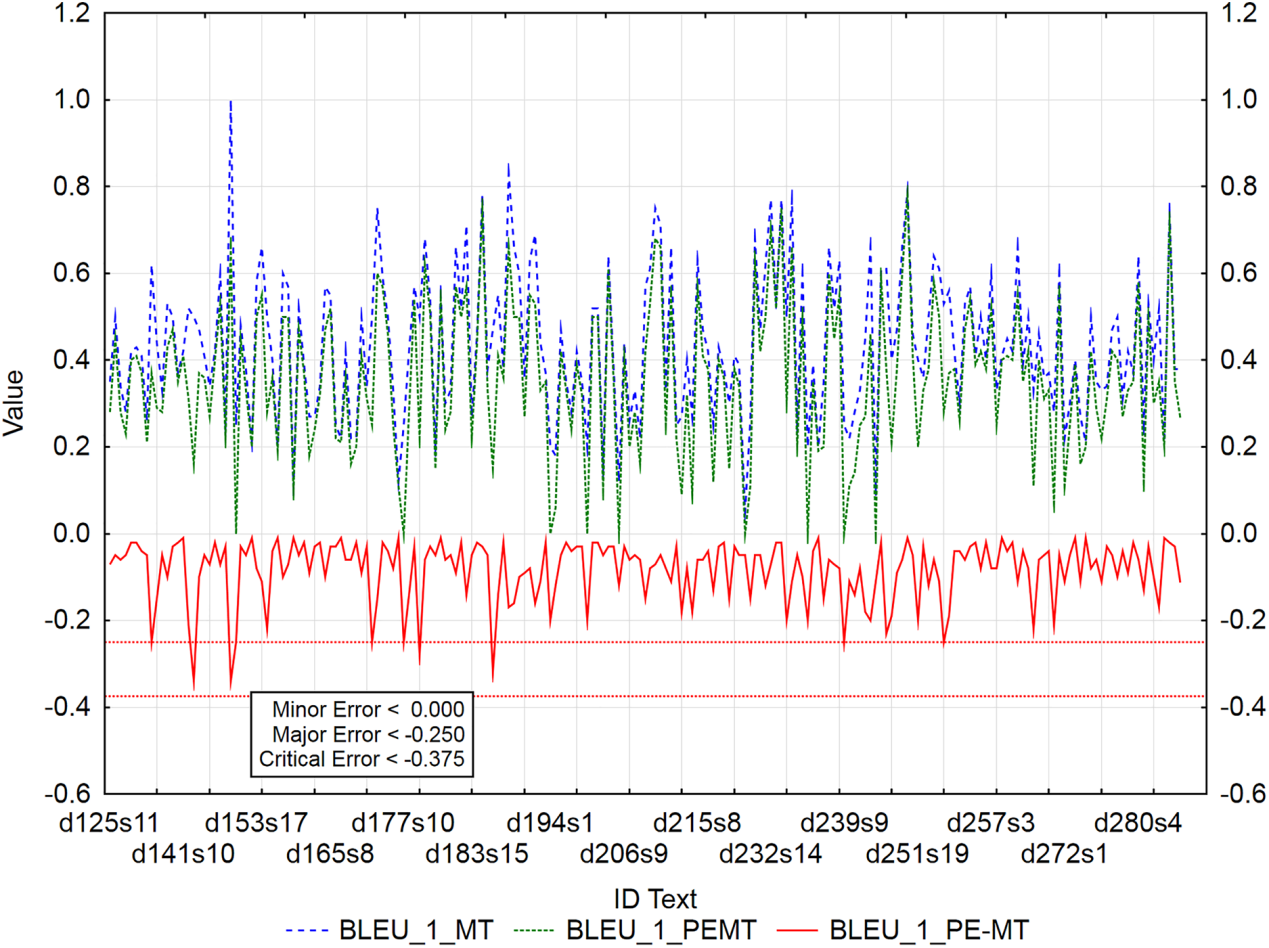
Identification of post-editing errors based on residuals of the metric BLEU_1 of PEMT and MT output.

**Figure 4:**
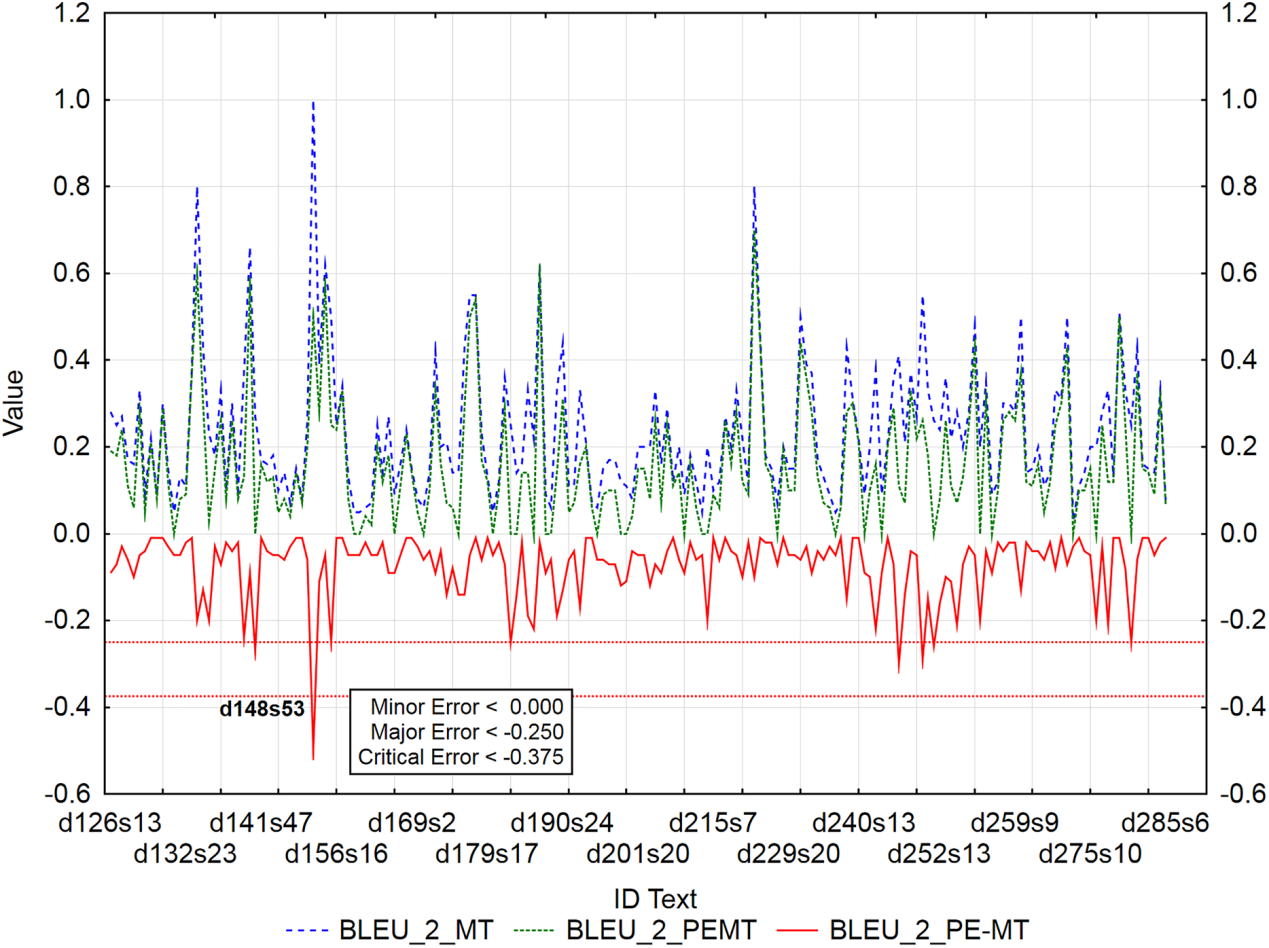
Identification of post-editing errors based on residuals of the metric BLEU_2 of PEMT and MT output.

**Figure 5:**
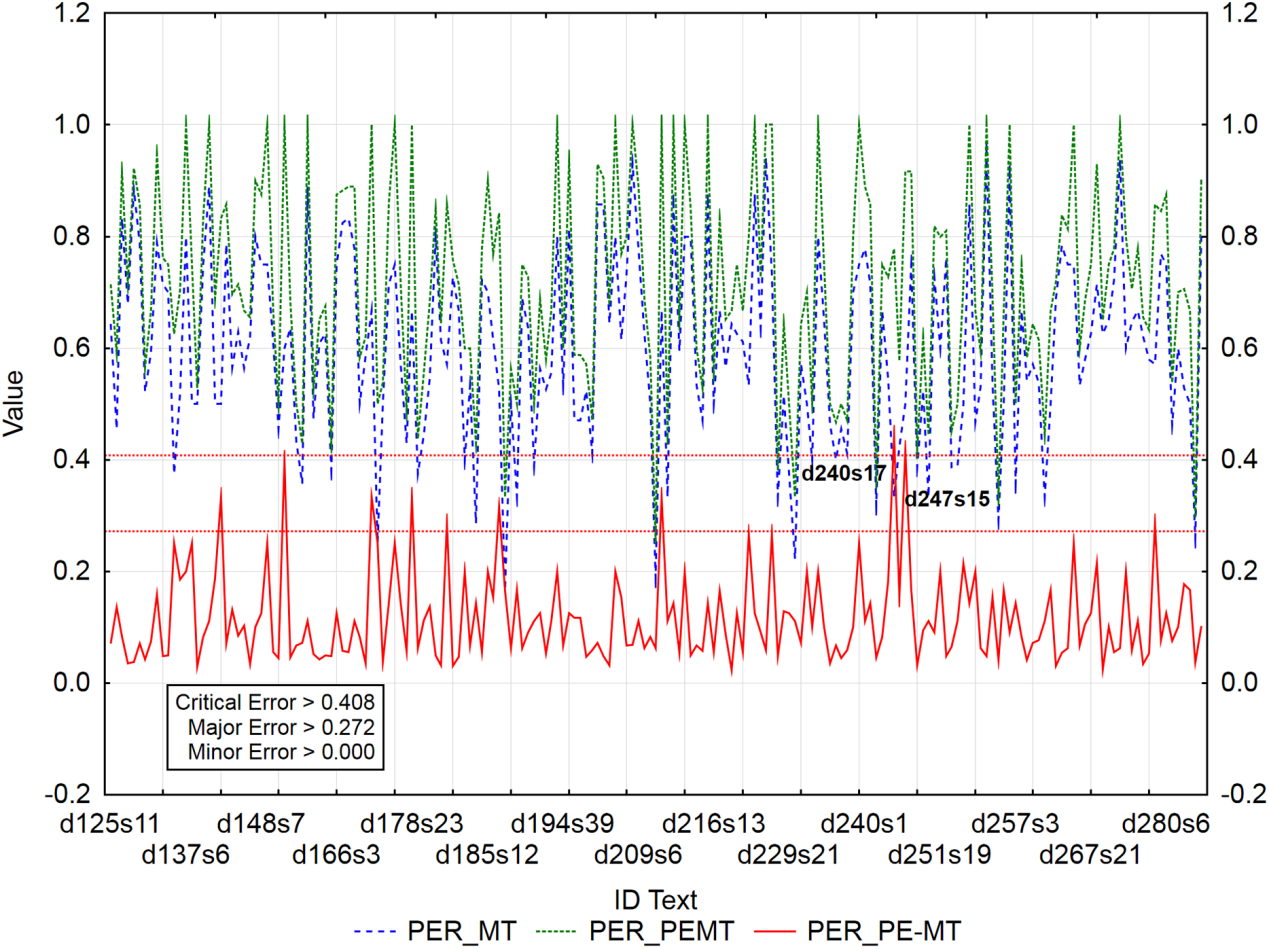
Identification of PE errors based on residuals of the metric PER of PEMT and MT output.

The graphs (Figs. 3–5) visualize the error rate metrics of the MT output (Err_MT), PEMT (Err_PEMT) and their residuals (Err_PE-MT). The residual values of error rate metrics (Err_PE–MT) of the PEMT and MT output identify segments (Figs. 3–5) with minor (> *Upper Quartile*), major *(> Upper Quartile + Quartile Range*), and critical error (> *Upper Quartile +* 1.5*Quartile Range*).

By residuals of the metric PER of PEMT and MT output (Fig. 3), from all examined 3,192 segments, 179 segments with a minor error, 8 segments with a major error and 2 segments with a critical error were identified.

The metrics WER and TER were identified as redundant, as they, along with the metric HTER, were highly consistent in identifying segments with minor, major, and critical errors (Table 12). This was also proved by the numbers of identified segments using the residuals of the metrics HTER/WER/TER of PEMT and MT output (Fig. 4). 186/184/185 segments with a minor error, 7/7/7 segments with a major error, and 2/2/2 segments with a critical error were identified out of a total of 3,192 segments (compare Data Matrix and Code).

In the case of residuals of the metrics PER and HTER, there was a 100% match in the identification of segments with a critical error (Figs. 3 and 4), namely, it is the 17th sentence in the document with ID 240 (d240s17) and the 15th sentence in the document with ID 247 (d247s15). On the contrary, in the case of segments with a minor error, the match was less than 50% and in the case of segments with a major error, less than 40% (Table 12). Among the error rate metrics, the metric CDER differed the most in identifying error segments. Using the residuals of the metric CDER of PEMT and MT output (Fig. 5), 166 segments with a minor error, 7 segments with a major error and 2 segments with a critical error were identified out of a total of 3,192 segments. The degree of concordance with other error rate metrics in identifying segments with a minor error was less than 50% and with a major error, less than 30% (Table 12). There was no match for segments with a critical error (Table 12, Figs. 3–5). Using residuals of the metric CDER (Fig. 5), sentences 7 and 9 in the document with ID 168 (d168s7 and d168s9) were identified as segments with a critical error (compare Data Matrix and Code).

**Figure 6:**
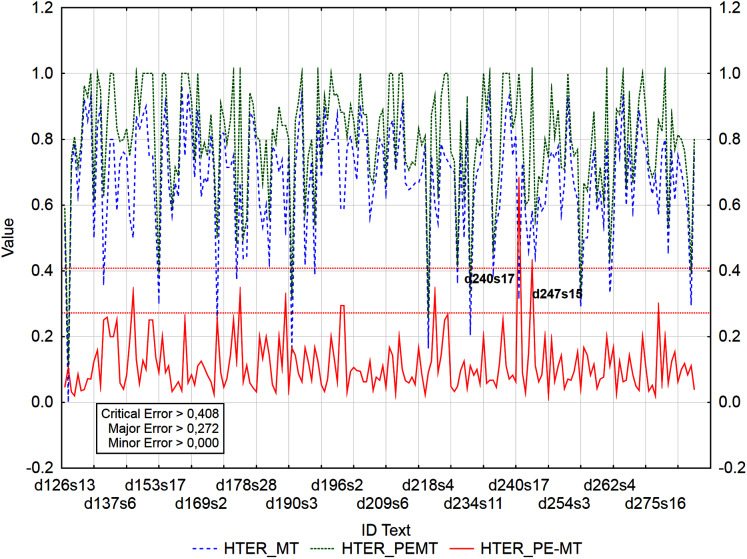
Identification of PE errors based on residuals of the metric HTER of PEMT and MT output.

**Figure 7:**
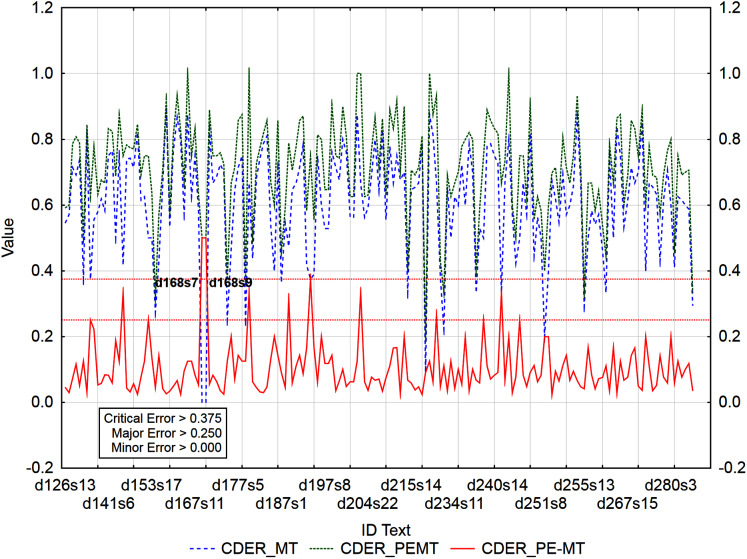
Identification of PE errors based on residuals of the metric CDER of PEMT and MT output.

**Figure 8:**
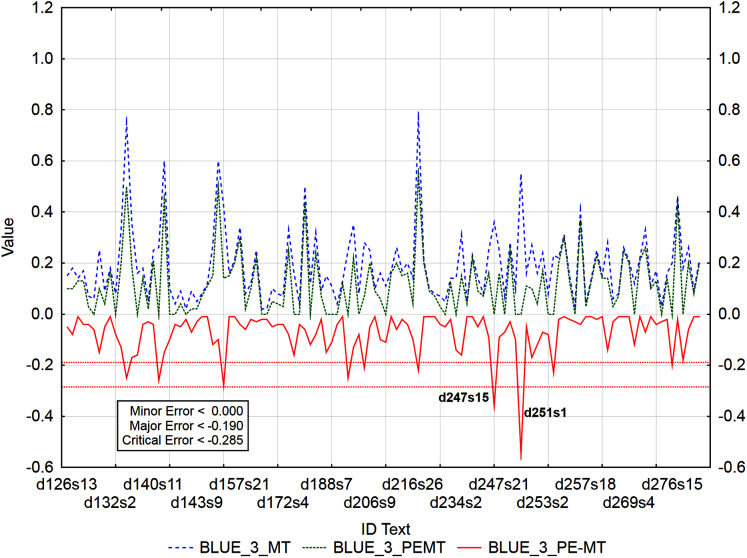
Identification of post-editing errors based on residuals of the metric BLEU_3 of PEMT and MT output.

**Figure 9:**
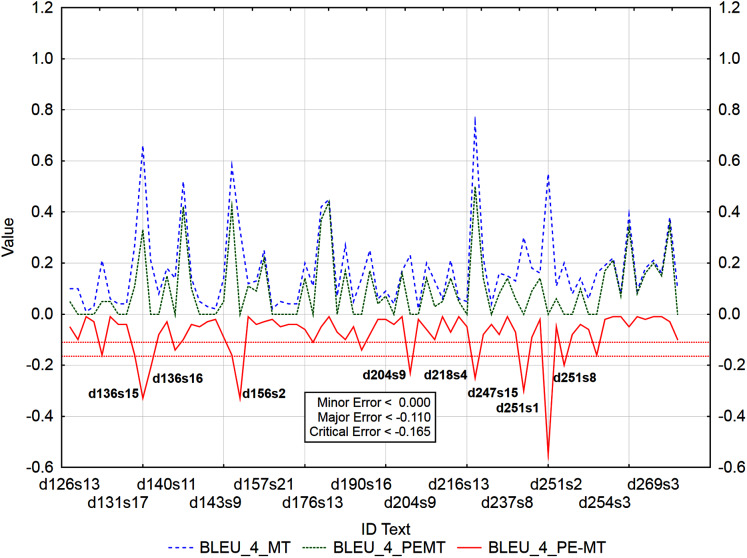
Identification of post-editing errors based on residuals of the metric BLEU_4 of PEMT and MT output.

Graphs (Figs. 6–9) visualize accuracy metrics of MT output (Cor_MT), PEMT (Cor_PEMT) and their residuals (Cor_PE-MT). The residual values of accuracy metrics of the PEMT and MT output (Cor_PE – MT) identify segments (Figs. 6–9) with minor (*< Lower Quartile*), major (*< Lower Quartile - Quartile Range*), and critical error (*< Lower Quartile –* 1.5 *Quartile Range*).

Out of 3,192 segments, 201 segments with a minor error and 4 segments with a major error and no segment with a critical error were identified using the residuals of metric BLEU_1 of PEMT and MT output (Fig. 6).

Similarly, 178 segments with a minor error, 4 segments with a major error and 1 segment with a critical error were identified by the residuals of the metric BLEU_2 of PEMT and MT output (Fig. 7), only the 53rd sentence in the document with ID 148 was identified to be critical (d148s53).

Out of 3,192 segments, using residuals of the metric BLEU_3 of PEMT and MT output (Fig. 8), 108 segments with a minor error, 8 with a major error, and 2 with a critical error were identified. Sentences 15 and 1 in documents with ID 247 and 251 (d247s15, d251s1) were identified as segments with critical errors (compare Data Matrix and Code).

Similarly (Fig. 9), 62 segments with a minor error, 6 segments with a major error, and as many as 8 segments with a critical error were identified out of 3,192 segments by residuals of the metric BLEU_4 of PEMT and MT output (d136s15, d136s16, d156s2, d204s9, d218s4, d247s15, d251s1, and d251s8). The largest matches in the identified segments (Table 13) were achieved for the residuals of the metrics BLEU_3 and BLEU_4. There were 43% matches in segments with a minor error, 27% matches in segments with a major error and 25% matches in segments with a critical error (compare Data Matrix and Code).

## Discussion

At present, there are no educational systems for teaching translation that are directly tailored to the needs of the student-translator providing the interaction. Olohan & Salama-Carr (2011) claim that a translator is no longer a solo player in the translation process but has a “machine” partner, which constitutes an agency with him/her. It also changes their mutual interaction into a “dance of agency” or even a struggle for this agency. However, a question arises concerning translator training for the future, *i.e.*, to prepare not solo agents, but translators, who would share their agencies with the “machine”.

When designing our system, we tried to fill the existing gap in online education (online translator training). The system is designed to keep an enrolled student in permanent interaction with a teacher or classmate, who both check his/her translation or PE quality. Moreover, it allows a mutual assessment of the translation or PE quality among classmates. This interaction can be synchronous or asynchronous.

Students can set their own learning pace and can work anywhere and at any time. Working with the online system the teacher/tutor can analyse a greater volume of translated or PEMT texts and cover the issues of the whole class, not only those of an individual. Students develop their translation competence and gain more experience in identifying PE errors.

When analysing a text, teachers may, for example, pay more attention to critical errors in PE. In the case of our research, 17 segments of critical errors were identified. After analysing 17 segments, we came to the conclusion, that:

(1) students tended to expand the texts, they tended to explain the context, *i.e.*, to insert extra words which were not in the source text. Students perceived the recipient of the translation as a person who had a different information base, and therefore students-translators added various explanations, introductions, afterwards, comments, etc. to the translation or PEMT (compare Data Matrix and Code).

For example: ID_d156s2 (Table 14) was identified as critical error (< −0.165) based on the residual of BLEU_4 metric (PEMT = 0, MT = 0.33, residual = −0.33), as major error (< −0.190) based on the residual of BLEU_3 metric (PEMT = 0.14, MT = 0.42, residual = −0.28), and as minor error (<0.000 and/or >0.000) based on the residuals of BLEU_1, BLEU_2, and CDER metrics. Based on metrics BLEU_1 to BLEU_4, we found that students had a need to insert extra words and keep the passive voice from the source language (English) in translation (Slovak).

**Table 14:** A detailed look at the segment ID_d156s2.

ST:	*Is it true climate change is making spiders bigger?*
MT:	*Je pravda, že zmena klímy je robiť pavúkmi väčšie?*
PEMT:	*Je pravda, že kvôli zmene klímy sa pavúky zväčšujú?*
TT:	*Je pravda, že zmena klímy zväčšuje pavúky?*

**Note:**

ST, source text; MT, machine translation; PEMT, post-edited MT; TT, target text.

For example: ID_d240s17 (Table 15) was identified as critical error (>0.408 and/or >0.272) based on the residuals of PER (PEMT = 0.778, MT = 0.333, residual = 0.445) and HTER metrics (PEMT = 1, MT = 0.333, residual = 0.667), as major error (>0.250) based on the residual of CDER metric (PEMT = 0.667, MT = 0.333, residual = 0.334) and as minor error (<0.000) based on the residuals of BLEU_1, BLEU_2, and BLEU_3 metrics. Based on metric PER, we found that students added extra words to the translation.

**Table 15:** A detailed look at the segment ID_d240s17.

ST:	*Many of those paths through the forest are hundreds of years old*.
MT:	*Mnohé z týchto ciest lesom sú stovky rokov*.
PEMT:	*Mnohé z týchto ciest vedú cez les a sú stovky rokov staré*.
TT:	*Mnohé z týchto lesných tratí sú staré stovky rokov*.

**Note:**

ST, source text; MT, machine translation; PEMT, post-edited MT; TT, target text.

(2) Secondly, they tended to use a considerable number of anglicisms in translation, although the analysed expression had an accurate equivalent in the target language (compare Data Matrix and Code).

For example: ID_d148s53 (Table 16) was identified as critical error (< −0.250) based on the residual of BLEU_2 (PEMT = 0.5, MT = 1, residual = −0.5) and as major error (< −0.250) based on the residual of BLEU_1 metric (PEMT = 0.66, MT = 1, residual = −0.34).

**Table 16:** A detailed look at the segment ID_d148s53.

ST:	*Super, super, cool*.
MT:	*Super, super, super*.
PEMT:	*Super, super, cool*.
TT:	*Super, super, skvelá*.

**Note:**

ST, source text; MT, machine translation; PEMT, post-edited MT; TT, target text.

Preferably it was suggested that the expressions should be used in their natural form and not in their origin or domestic forms.

Similarly, we focused on major and minor PE errors. Within the major errors, mainly synonyms and declensions were identified (compare Data Matrix and Code).

For example: ID_d197s8 (Table 17) was identified as major error (>0.272) based on the residual of HTER (PEMT = 0.882, MT = 0.588, residual = 0.294) and as minor error (<0.000 and/or >0.000) based on the residuals of BLEU_2 metric (PEMT = 0.1, MT = 0.17, residual = −0.07), CDER (PEMT = 0.647, MT = 0.529, residual = 0.118), and PER (PEMT = 0.588, MT = 0.471, residual = 0.117).

**Table 17:** A detailed look at the segment ID_d197s8.

ST:	*“Often you can’t see it or smell it, but it’s there – and air pollution is risking the health of an entire generation of children.”*
MT:	*"Často nemožno vidieť ani cítiť, ale je to tam. - A znečistenie ovzdušia riskuje zdravie celé generácie detí"*
PEMT:	*"Často ho nie je vidieť ani cítiť, ale existuje. - A znečistenie ovzdušia je zdravotným rizikom pre celé generácie detí"*
TT:	*"Znečistenie často necítime ani nevidíme, ale je všade - a predstavuje riziko pre zdravie celej generácie detí."*

**Note:**

ST, source text; MT, machine translation; PEMT, post-edited MT; TT, target text.

For example: ID_d141s41 (Table 18) was identified as major error (< −0.250/>0.272/>0.250) based on the residuals of BLEU_2 (PEMT = 0, MT = 0.27, residual = −0.27), BLEU_1 (PEMT = 0.16, MT = 0.5, residual = −0.34), PER (PEMT = 0.833, MT = 0.5, residual = 0.333), CDER (PEMT = 0.75, MT = 0.417, residual = 0.333), and HTER (PEMT = 0.833, MT = 0.5, residual = 0.333), and as minor error (<0.000) based on the residual of BLEU_3 metric (PEMT = 0, MT = 0.1, residual = −0.1).

**Table 18:** A detailed look at the segment ID_d141s41.

ST:	*Try it at Figueira Rubaiyat, a beautiful restaurant built around an enormous fig tree*.
MT:	*Skúste si to na Figueira Rubaiyat, krásne reštaurácie postavený okolo obrovského figovníka*.
PEMT:	*Skúste Figueira Rubaiyatu, krásnu reštauráciu postavenú okolo obrovskej figy*.
TT:	*Objednajte si ho vo Figueira Rubaiyat, nádhernej reštaurácii postavenej okolo obrovského figovníka*.

**Note:**

ST, source text; MT, machine translation; PEMT, post-edited MT; TT, target text.

Minor errors were related to typos, word order, punctuation and abbreviations. However, these errors are easily eliminated or removed (compare Data Matrix and Code).

For example: ID_d223s21 (Table 19) was identified as minor error (>0.000) based on the residual of HTER metric (PEMT = 0.636, MT = 0.545, residual = 0.091).

**Table 19:** A detailed look at the segment ID_d223s21.

ST:	*William T. Terrell told us *via* Facebook about one such example:*
MT:	*William T. Terrell nám cez Facebook o jeden taký príklad:*
PEMT:	*William T. Terrell nám o jednom takom prípade povedal cez Facebook*.
TT:	*William T. Terrell nám prostredníctvom Facebooku povedal o jednom takom prípade:*

**Note:**

ST, source text; MT, machine translation; PEMT, post-edited MT; TT, target text.

The research findings fully correspond with the student's achievements during the official final examination. When being examined their task was to translate certain text from a foreign language into their mother tongue within a limited time. To assure objectivity during the final examination, two different teachers assessed the quality of their translations as each teacher had a different sensitivity to error rate. Certainly, this type of evaluation was highly labour intensive and time-consuming. Although both approaches, automatic (based on residuals) and traditional-manual, achieved approximately the same results, a different level of effort was required. While automatic evaluation of translation quality still requires human intervention, the extent thereof is different.

As Doherty (2017) claims evaluation of translation quality is crucial not only in the educational context as a formative assessment of the translator, but also as part of MT research, its quality and translator productivity.

## Conclusions

Due to time and financial concerns and the currently exacerbated epidemiological situation associated with COVID-19, MT and PE in the form of subtitles have become widely applied to online educational contexts.

The study offers new insight into translator training online. The results and findings of the research offer two key theoretical contributions and two practical contributions to the field of translator training online.

The first theoretical contribution consists of the design and verification of an approach for assessing students’ translation competences. Firstly, we verified that the residuals of accuracy (BLEU_n) and error rate (PER, WER, TER, CDER and HTER) of PEMTs and MT outputs could be used to assess students´ translation competence (validity). We verified that the residuals are valid for student assessment. As a criterion for validity, the results of the final examination taken at the end of the bachelor study were considered (grades A, B, C, D, or E).

Subsequently, the second theoretical contribution comprises the boundaries or limits for minor, major or critical errors identification in PEMTs based on residuals. Only two residuals of metrics of the error rate were identified as redundant (WER and TER) and none of the metrics of accuracy.

From a practical point of view, the findings offer a closer understanding of those errors that arise in PE. It consists of the identification of the segments for each error category (minor, major, and critical) that teachers should address or pay attention to. When teaching translation, the teacher could focus on all the students’ PEMTs (in this case 3,192 segments), *i.e.*, it was not necessary to analyse the whole text, sentence by sentence (3,192 segments), but only segments with critical (17 segments), major (44 segments), or minor errors. Attention was paid to 170 problematic textual segments (including segments with minor errors) of a given journalistic genre and a selected group of students. Based on the results, it is possible to predict tendencies in the error rate during PE and to analyse them. On the other hand, the teacher could focus not only on the whole students’ group in general but could also pay attention to a particular student, to her/his performance during the translation, *i.e.*, the teacher could take a particular learner’s needs into account and set or tailor the specific needs into the lesson. This is one of the goals of teaching and learning online and online educational contexts in general.

The second practical contribution lies in the creation of the unique OSTPERE system, a system for teaching and learning translation and PE online, based on human-machine interaction, in which we teach students not only to translate and post-edit, but also to assess the quality of translation and PE. Moreover, the system also enables human-human interaction, in the form of the mutual evaluation of translations or PEMTs among students as well as an evaluation of translation quality by the teacher.

This approach to training and assessing students may increase the overall effectiveness of online teaching and learning, *e.g.*, translation and PE, and prevent the large dropout of students entering online education instead of accomplishing the planned goals within the course. As Murray (2019) stated, over a period of five years (2013–2018) up to 96 percent of students dropped out of online forms of study. It is possible that this was caused by ignoring students’ learning needs and preferences. Moreover, students, who serve an internship or stay abroad, could easily complete the course in the selected term. Similar questions were raised also by Al Abdullatif & Velazquez-Iturbide (2020).

The research also has certain limitations from the aspect of one small-size group—only 20 students, text styles and translation directions. For this reason, we want to focus our future work on text volume, extend the group-size, and reverse translation direction (from mother tongue to a foreign language) as well as a diversity of genres such as manuals, technical documentation, and administrative texts. Also, other techniques for data collection (*e.g.*, different automatic metrics of MT evaluation) could be employed to elicit more in-depth information. We would like to link automatic metrics and other predictors to the error typology we have developed (Munková et al., 2017). For this purpose, we will solve a classification task where the input variables will be automatic metrics and other predictors and the output will be error categories reflecting the created error typology of MT when translated into Slovak. It will be necessary to identify error categories that can be explained by the mentioned predictors. We are aware that based on models with a dominance of automatic evaluation metrics, it will not be possible to predict all error categories resulting from the created error typology.

In a future direction, we want to try to estimate the quality of machine translation without reference (so far, our results refer to reference). We plan to use machine learning to build a quality estimation model, focusing on estimating the quality of machine translation at the level of words (phrases) and sentences for a language pair—Slovak and English.

## Supplemental Information

10.7717/peerj-cs.706/supp-1 Supplemental Information 1 Evaluation of MTs and PEMTs using automatic metrics and their residuals. Raw data with calculated automatic metrics and their residuals

10.7717/peerj-cs.706/supp-2 Supplemental Information 2 Evaluation of MTs and PEMTs using automatic metrics and their residuals (alternate file). Raw data with calculated automatic metrics and their residuals (alternate file)

10.7717/peerj-cs.706/supp-3 Supplemental Information 3 Application code for automatic metrics calculation.

## Additional Information and Declarations

### Data Availability

The following information was supplied regarding data availability: The data matrix and the application code for automatic metrics calculation is available in the Supplemental Files.
